# Practical Notes and Formulæ

**Published:** 1882-10-20

**Authors:** 


### PRACTICAL NOTES AND FORMULÆ.

**For Tonsillitis.**—T. P. Jones, M. D., has found the following of service:

℞ Fl. ext. ergot........................................gtt xl.
Acid carbolic........................................gtt i.
Aquæ........................................℥ iss.

M. (Shake.) Sig.—For throat atomizer, spray every half hour.

If the tonsils are much swollen, a feeling as if something was pulling upon them is produced. This disagreeable feeling subsided after two or three times spraying.—*Therapeutic Gazette.*

**Iodine in Croupal Pneumonia.**—According to the author—in uncomplicated croupal pneumonia—iodine and iodide potassium are specified if given within 36 hours after the commencement of the attack. Of 98 cases treated, and 10 of them with details of the treatment, the result was very satisfactory. He considers that iodine destroys the diseased germs and aborts the disease rapidly. The following formulæ were used:

℞ Tr. iodine.............................. 5 drops.
Distilled water..............................120 grammes.

Dose—A large spoonful every hour. Iodide potassium 1½ grains in sweetened water every hour.—*Dr. Schwarts, in Revista Madrid.*

**To Remove Freckles.**—

℞ Oil of almonds, exp'd..........fl. ℥ iv; 120.00 fl. Gm

Lard..........................℥ iij; 90.00 Gm

Spermaceti....................℥ j; 30.00 Gm

Expressed juice of house-leek ...fl. ℥ iij; 90.00 fl. Gm

Melt the spermaceti and lard together; add the oil and then the juice and stir the mixture until it solidifies on cooling. A few drops of some perfume, as cologne, may be added.—*Oil and Drug News.*

**Chandler's Chlorodyne.**—

℞ Muriate of Morphia...................... 8 grains.
Fluid extract of cannabis indica..........30 minims.
Oil of peppermint......................10 drops.
Tr. of capsicum.........................15 drops.
Chloroform.............................. 2 drachms.
Alcohol.............................. 1 ounce.
Glycerine.............................. 1 "

Mix. Dose, ten to thirty drops in a wine glass of water.—*Druggists' Circular.*

**Mistura Opii Composita.**—At a recent pharmaceutical meeting of the Philadelphia College of Pharmacy, Prof. Maisch communicated a formula obtained from Dr. W. A. Hammond for the above mixture. It is made as follows:

℞ Fl. extract of coca........................2 ounces.
Fl. extract of viburnum....................1 ounce.
Fl. extract of opium graveolens..............1 ounce.

The mixture is designated Mistura Opii Composita for convenience, and forms an excellent nerve sedative and tonic. The dose is from one to two teaspoonfuls three times a day.—*Druggist's Circular.*

**A Sedative Emmenagogue.**—For a day or two antecedent to the actual commencement of the catamenial flux, women not infrequently suffer acute pain in the pelvic region, doubtless due to hyperæmia and hyperæsthesia of the reproductive belongings. To obviate this, I have found no treatment give such satisfactory results as the following:

℞ Codeiæ sulphatis..........................gr. j.

Chloral hydratis.........................} aa gr. xx

Ammonii bromidi ........................}

Acquæ camphoræ.........................℥ j.

M. For one dose. S. Take at bedtime.

A repetition of the dose at that period is rarely necessary. In some cases a warm sitz-bath of fifteen minutes duration before retiring is a valuable adjuvant.—*Western Med. Rep.*—*Vir. Med. Monthly, March, 1882.*

**A New Remedy for Tapeworm.**—According to Dr. H. Witt (St. Petersburg Medical Weekly Journal, April, 1882), the alkaloid pelletierinum, lately extracted from the bark of the root of the pomegranate, is the most certain of all remedies for tapeworm. In five cases where all other remedies had failed, about twenty-two grains of this drug, followed by a tablespoonful of castor oil, killed the worm, which appeared unbroken in the stool. The remedy is said to be perfectly tasteless.—*Therapeutic Gazette.*

**Salicylic Cream.**—Dr. Alexander Ogston (Med. Times and Gazette) recommends as a valuable agent for keeping sponges, tents, instruments, etc., aseptic in the vagina, the following:

℞ Acid salicylic (pulv.)....................1 part.
Glycerin or vaselin......................4 or 5 parts.

Dr. Matthews Duncan recently commended this preparation to the London Obstetrical Society, stating that "he had used it with success in inducing premature labor and other operations."

**Granular Eyelids.**—If, on eversion, the lids present that peculiar velvety appearance present in granular lids, use some astringent, such as sulphate of copper in crystal, or paint over the surface a solution of nitrate of silver, forty grains to the ounce of water. It might be well also to apply once or twice a day the following ointment:

| | | | |
|---|---|---|---|
| ℞ | Hydrarg. ox. rub. / Zinci carb. | aa gr. xv; | 1.00 Gm. |
| | Ext. opii | ℈j; | 1.33 Gm. |
| | Pulv. camphoræ | gr. iij; | 0.18 Gm. |
| | Ung. acquæ rosæ | ℥ ss; | 15.00 Gm. |

M. Sig. Rub in a small piece on the lids once or twice daily. *Med. Bulletin.*

**Mosquito Oil.**—

| | |
|---|---|
| Oil of tar | 1 ounce. |
| Olive oil | 1 ounce, |
| Oil of pennyroyal, | ½ ounce, |
| Spirit of camphor | ½ ounce, |
| Glycerine | ½ ounce, |
| Carbolic acid | 2 drachms. |

M. Shake well before using.—*Drug. Circular.*

**Membranous Dysmenorrhœa.**—Chlor. hydrate, pot. brom. aa ʒ ii; morh. sulph. grs. iss; Syrup aurcorticis, ℥ iii. Misci. Sig. A dessert spoonful in a wine glass of water every four hours while in pain.—*Thomas.*

**Cholera Pills.**—The following prescription was recommended highly by the late Dr. Chas. Meigs as a cholera pill:

| | | |
|---|---|---|
| ℞ | Morph. sulph | 13 grains, |
| | Camphoræ | 20 grains, |
| | Ol. cajeputi | 10 drops, |
| | Tragacanth | 5 grains, |
| | Ext. gent | 15 grains, |
| | Syrp. acaciæ, q. s. | |

M. Ft. Mass. et div. pil. No. 100.—*Med. Bulletin.*

**Neuralgia.**—Dr. David L. Wallace, of Newark, N. J., recommends the following ointment for neuralgia through the columns of the Med. Record:

| | | |
|---|---|---|
| ℞ | Veratriæ | 4 grains, |
| | Morphiæ | 6 grains, |
| | Tr. aconit. rad. | 1½ dracqms, |
| | Vaselinæ | 1 ounce. |

M. Sig. To be applied to painful part every fifteen minutes.—*Peoria Med. Monthly.*

**For Bites of Fleas. Etc.**—The most agreeable and effective application to the skin is a tincture of the pyrethum roseum, made from the powder, shaken up in Eau de Cologne.—*Med. So. Co. of Kings.*

